# Preclinical Evaluation of the Novel, Orally Bioavailable Selective Inhibitor of Nuclear Export (SINE) KPT-335 in Spontaneous Canine Cancer: Results of a Phase I Study

**DOI:** 10.1371/journal.pone.0087585

**Published:** 2014-02-04

**Authors:** Cheryl A. London, Luis Feo Bernabe, Sandra Barnard, William C. Kisseberth, Antonella Borgatti, Mike Henson, Heather Wilson, Kiersten Jensen, Daisuke Ito, Jaime F. Modiano, Misty D. Bear, Michael L. Pennell, Jean-Richard Saint-Martin, Dilara McCauley, Michael Kauffman, Sharon Shacham

**Affiliations:** 1 Departments of Veterinary Biosciences and Veterinary Clinical Sciences, College of Veterinary Medicine, The Ohio State University, Columbus, Ohio, United States of America; 2 Department of Veterinary Clinical Sciences and Masonic Cancer Center, University of Minnesota, Minneapolis/St. Paul, Minnesota, United States of America; 3 Department of Small Animal Clinical Sciences, Texas A&M University, College Station, Texas, United States of America; 4 Division of Biostatistics, College of Public Health, The Ohio State University, Columbus, Ohio, United States of America; 5 Karyopharm Therapeutics, Natick, Massachusetts, United States of America; University of North Carolina at Chapel Hill, United States of America

## Abstract

**Background:**

The purpose of this study was to evaluate the activity of Selective Inhibitors of Nuclear Export (SINE) compounds that inhibit the function of the nuclear export protein Exportin 1 (XPO1/CRM1) against canine tumor cell lines and perform a Phase I clinical trial of KPT-335 in dogs with spontaneous cancer to provide a preliminary assessment of biologic activity and tolerability.

**Methods and Findings:**

Canine tumor cell lines derived from non-Hodgkin lymphoma (NHL), mast cell tumor, melanoma and osteosarcoma exhibited growth inhibition and apoptosis in response to nanomolar concentrations of SINE compounds; NHL cells were particularly sensitive with IC_50_ concentrations ranging from 2–42 nM. A Phase I clinical trial of KPT-335 was performed in 17 dogs with NHL (naive or relapsed), mast cell tumor or osteosarcoma. The maximum tolerated dose was 1.75 mg/kg given orally twice/week (Monday/Thursday) although biologic activity was observed at 1 mg/kg. Clinical benefit (CB) including partial response to therapy (PR, n = 2) and stable disease (SD, n = 7) was observed in 9/14 dogs with NHL with a median time to progression (TTP) for responders of 66 days (range 35–256 days). A dose expansion study was performed in 6 dogs with NHL given 1.5 mg/kg KPT-335 Monday/Wednesday/Friday; CB was observed in 4/6 dogs with a median TTP for responders of 83 days (range 35–354 days). Toxicities were primarily gastrointestinal consisting of anorexia, weight loss, vomiting and diarrhea and were manageable with supportive care, dose modulation and administration of low dose prednisone; hepatotoxicity, anorexia and weight loss were the dose limiting toxicities.

**Conclusions:**

This study provides evidence that the novel orally bioavailable XPO1 inhibitor KPT-335 is safe and exhibits activity in a relevant, spontaneous large animal model of cancer. Data from this study provides critical new information that lays the groundwork for evaluation of SINE compounds in human cancer.

## Introduction

The exchange of proteins between the nucleus and cytoplasm is a tightly regulated process that involves several proteins responsible for shuttling cargo in and out of the nucleus. There are seven known nuclear export proteins (Exportin 1–7) that carry macromolecules from the nucleus to the cytoplasm [1], [2], [3]. Exportin 1 (XPO1, also called Chromosome Region Maintenance protein 1 [CRM1]) is a member of the karyopherin β family of transport receptors that binds a very diverse set of approximately 220 target proteins through a hydrophobic leucine-rich nuclear export signal (NES) present in the cargo [4]. Interaction of the NES-directed protein cargo with the small GTPase molecule Ran leads to cytoplasmic transport via a nuclear pore complex [5]. XPO1 is the sole nuclear exporter of several major tumor suppressor and growth regulatory proteins (TSPs and GRPs, including p53, p75, Rb, p21, p27, STAT3, FOXO and IκB among others) [6], [7]. Expression of XPO1 is known to be upregulated in a variety of both hematologic malignancies and solid tumors and this correlates with a poor prognosis [1], [2], [3], indicating that changes in nuclear-cytoplasmic trafficking resulting in aberrant localization of key proteins can contribute to tumorigenesis and potentially resistance to therapy.

Several small molecule inhibitors of XPO1 have been developed and tested against a variety of neoplastic cells, primarily *in vitro*. These include Leptomycin B, ratjadone, anguinomycin, goniothalamin, among others, that bind covalently to a reactive cysteine residue (Cys528) located in the NES-binding groove of XPO1 [1], [2], [3]. This binding functionally inactivates XPO1 in an irreversible manner and targets the protein for proteasome degradation [8], resulting in restoration of TSP and GRP cellular localization and function. For example, Leptomycin B causes nuclear retention of the BCR-ABL1 fusion protein and induces apoptosis when co-administered with imatinib to CML cells *in vitro* [9]. While Leptomycin B exhibits activity against several cancer cell lines *in vitro* and mouse xenograft tumor models, it induces significant systemic toxicity in both animals and humans resulting in discontinuation of clinical development [10]. More recently, analogs of Leptomycin B have been developed that demonstrate greater potency *in vitro* and *in vivo* with a significantly reduced toxicity profile in mice [11]. However, these agents require intravenous delivery, limiting the frequency of drug administration.

Recently, novel, drug-like, orally bioavailable, small-molecule Selective Inhibitor of Nuclear Export (SINE) compounds that specifically and irreversibly bind to XPO1 at the reactive site Cys 528 residue have been developed [12], [13], [14], [15], [16], [17], [18]. These are slowly reversible with a t_1/2_ of approximately 24 hours, and lead to functional inactivation the XPO1 protein. SINE compounds have been shown to induce apoptosis and block proliferation in several cancer cell lines, including those derived from colon [6], pancreas [12], and breast carcinomas [16] as well as chronic lymphocytic leukemia (CLL) [15], while sparing normal cells [19]. Additional studies have shown potent anti cancer activity and good tolerability of SINE *in vivo* using mouse human xenograft (subcutaneous, orthotopic, or leukemograft) models of pancreatic cancer [12], renal cancer [20], CLL [15], mantle cell lymphoma (MCL) [18], multiple myeloma [8] and acute myelogenous leukemia (AML) [17]. These data support the notion that SINE compounds will have biologic activity in humans with cancer.

Spontaneous canine cancers exhibit many clinical and molecular similarities to human cancers and as such, serve as an attractive model for preclinical studies that evaluate the biologic activity and toxicities of novel anti-cancer therapeutics. Such studies have been used to validate the activity of multi-targeted receptor tyrosine kinase inhibitors (toceranib) [21], [22], new chemotherapeutic agents (GS-9219) [23], and various other small molecule inhibitors (ibrutinib, STA-1474) [24], [25]. Therefore, the purpose of this work was to examine the effects of SINE compounds in canine spontaneous cancers. Specifically, their activity was first assessed *in vitro* against canine tumor cell lines with a specific emphasis on hematopoietic tumors, after which a Phase I study of the novel SINE KPT-335 was performed in dogs with metastatic osteosarcoma, mast cell tumor and non-Hodgkin lymphoma (NHL). These studies laid the groundwork for the current Phase I evaluation of the SINE compound KPT-330 in humans with cancer and for a potentially pivotal study of KPT-335 for dogs with newly diagnosed or relapsed NHL.

## Materials and Methods

### Primary Tumor Samples and Cell Culture

Cryopreserved tumor cells obtained from sterile lymph node biopsy samples from dogs with diffuse large B cell lymphoma (DLBCL) were cultured with 100 ng/mL of megaCD40L (Enzo Life Science, Plymouth Meeting, PA) as previously described [26], [27]. The human T-cell leukemia cell line, Jurkat, was from the American Type Culture Collection (ATCC, Manassas, VA) and cultured in RPMI1640 medium (Gibco/BRL, Grand Island, NY) containing 10% fetal bovine serum (FBS; Atlas Biologicals, Fort Collins, CO), 2-mercaptoethanol (Gibco/BRL), HEPES, L-glutamine, sodium pyruvate (Mediatech Inc., Manassas, VA), non-essential amino acids (Sigma Aldrich, St. Louis, MO), and Primocin (Invivogen, San Diego, CA). The canine DLBCL cell line CLBL1 obtained from Dr. Barbara Rütgen (University of Vienna, Austria) [28], [29] and human DLBCL cell lines OCI-Ly3 [30], [31] and OCI-Ly10 [32] obtained from Dr. Anne Novak (Mayo Clinic Cancer Center, Rochester, MN) were cultured in complete Iscove’s Modified Dulbecco’s medium (IMDM) containing 20% FBS, L-glutamine, and Primocin. All cells were maintained at 37°C in a humidified 5% CO_2_ atmosphere. The following additional canine tumor cell lines were used to evaluate response to SINE compounds: C2, mast cell tumor line provided by Dr. George Caughey, UCSF, San Francisco, CA [33]; OSA16, osteosarcoma cell line, provided by Dr. Jaime Modiano, UM, Minneapolis, MN [34]; and 323610-3, malignant melanoma cell line, provided by Dr. Michael Kent, UCD, Davis, CA [35]. All cell lines were cultured in complete RPMI media containing 10% FBS, antibiotic/antimycotic, HEPES, sodium pyruvate, nonessential amino acids and Glutamax (media supplements from Gibco).

### Viability and Proliferation Assays

Cell viability for lymphoid lines was determined by the MTS (3-(4, 5-dimethylthiazol-2-yl)-5-(3-carboxymethoxyphenyl)-2-(4-sulfophenyl)-2H-tetrazolium) assay using CellTiter 96® AQ_ueous_ One Solution Cell Proliferation Assay Kit (Promega, Madison, WI). Briefly, for lymphoid cell lines, 5×10^4^ cells (or 1×10^5^ primary DLBCL cells) were cultured in 100 µL of complete medium in 96-well plates in the presence of SINE compounds. After 72 hours, 20 µL of MTS solution was added to each well and cells were incubated for another 4 hours before measuring absorbance at 490 nm using a Wallac Victor 1420 Multilabel Counter (Perkin Elmer, Waltham, MA). The IC_50_ of SINE was calculated using Prism 6 software (GraphPad Software, Inc., La Jolla, CA).

For the non-lymphoid cell lines, 96 well plates were seeded in triplicate in 90 µL with 2500 cells/well of OSA16, 5000 cells/well of C2, and 2500 cells/well of 323610-3. Seeded plates were cultured overnight then treated the following day with 10 µL of KPT-214 in C10 media at concentrations of 0.0001, 0.01, 0.1, 1.0, and 10 µM. Plates were collected at 92 hours, centrifuged at 1300 rpm, and supernatant was removed by inverting plates on absorbent paper. Plates were then sealed and immediately placed at −80°C for a minimum of 12 hours. Plates were then thawed and CyQUANT ®Cell Proliferation Assay (Life Technologies) was performed following the manufacturer’s protocol. Briefly, 200 µL of the diluted working CyQUANT solution was added to each well and protected from light. Fluorescence was the measured using a SpectraMax M2 microplate reader at 480 nm excitation and 520 nm emission. Results were represented as percent of control, or plotted to calculate IC_50_ values at 92 hours.

### Apoptosis Assay

Jurkat cells and primary canine DLBCL cells were cultured for 24 hours in the presence of 100 nM KPT-335 or dimethyl sulfoxide (DMSO). Cells were stained with Annexin V (eBiosciences, San Diego, CA) according to the manufacturer’s instruction. Flow cytometry was performed using a BD LSRII flow cytometer (BD Immunocytometry Systems, San Jose, CA) and results were analyzed using FlowJo software (Tree Star, Ashland, OR).

### Immunoblotting

Cryopreserved primary canine DLBCL and CLBL1 cells were lysed in RIPA buffer containing 150 mM NaCl, 50 mM Tris, pH 7.4, 0.1% sodium dodecyl sulfate (SDS), 1.0% Triton X-100, 1.0% sodium deoxycholate, 5 mM EDTA, 1 mM dithiothreitol, and a Proteinase Inhibitor Cocktail (Sigma-Aldrich, St. Louis, MO). Insoluble material was removed by centrifugation, and protein concentrations of the cell lysates were determined using the BioRad Protein Assay kit (BioRad, Hercules, CA). Proteins (100 µg) were separated by SDS-polyacrylamide gel electrophoresis (SDS-PAGE) and transferred to nitrocellulose membranes (BioRad, Hercules, CA). Antibody staining was performed using the SNAP i.d. system (EMD Millipore, Billerica, MA) according to the manufacturer’s instructions. Briefly, membrane was blocked by 50% LI-COR blocking buffer (LI-COR, Lincoln, NE) and stained with rabbit anti-XPO1 antibody (1∶66 dilution, Santa Cruz, Santa Cruz, CA) and mouse anti-actin antibody (1∶1666 dilution, Sigma-Aldrich), followed by secondary donkey anti-rabbit antibody conjugated to IRDye800 and anti-mouse antibody conjugated to IRDye680 (1∶3,333 dilution, LI-COR). Detection was performed using the Odyssey Infrared Imaging System (LI-COR).

### KPT-335 Formulation

KPT-335 was prepared as gelatin filled capsules in strengths ranging from 2.5 mg to 20 mg of active pharmaceutical ingredient (API). The API was wet milled with Lutrol F68 NF (BASF) and water in a Microfluidics micro-fluidizer, combined with Plasdone K29/30 (ISP Technologies), and lyophilized. Lyophilized powders contained 70–75% API. Capsules were individually hand filled with lyophilized powder to the desired dosage strength. All excipients were of suitable grades for use in pharmaceuticals.

### Pharmacokinetic Analysis in Healthy Dogs

KPT-335 was administered as a single oral dose in capsule formulation to 6 male beagle dogs at approximately 1.5 mg/kg following a meal. Serial blood samples were collected from each dog prior to dosing and at 0.25, 0.5, 1, 2, 4, 6, 8, 12, 18, 24 and 48 hours post-dose and placed into tubes containing K_2_EDTA. Blood samples were stored on wet ice until processed to plasma by centrifugation at 3500 rpm for 10 minutes at 5°C. Plasma samples were stored in at −80°C until analysis at Agilux Laboratories (Worcester, MA). Plasma samples were analyzed for parent drug concentration using sample preparation by protein precipitation and then analysis by LC-MS/MS using propranolol as the internal standard. The method was qualified for use (a single batch to assess method performance: precision and accuracy; linearity of dilution; and matrix specificity) prior to initiation of sample analysis. Acceptance criteria for calibration standards and quality control samples were within ±30% nominal concentration. Individual animal pharmacokinetic parameter values were derived by the pharmacokinetic analysis program Phoenix WinNonlin version 6.3 (Pharsight Corporation), using the non-compartmental Model 200 with uniform weighting. The single-dose pharmacokinetic parameters assessed include: C_max_ (maximum concentration observed); T_max_ (time of observed maximum concentration); AUC_0→∞_ (area under the concentration-time curve from time zero extrapolated to infinity); AUC_0→last_ (area under the concentration-time curve from time zero to the time of the last quantifiable concentration); and t_1/2_ (terminal half life). Descriptive statistical data (mean, standard deviation, and standard error of the mean) were calculated from the unrounded numbers in an Excel (Microsoft) spreadsheet. Concentration results, means, and calculated parameters were reported to 3 significant figures.

### Clinical Trial Eligibility

This clinical trial was approved by the Ohio State University (OSU) Veterinary Medical Center (VMC) Clinical Research Advisory Committee and the OSU IACUC; IACUC approval was also obtained at University of Minnesota and Texas A&M University. Written informed consent from the owner of each dog was obtained prior to study entry. KPT-335 was administered to dogs with metastatic osteosarcoma, mast cell tumor or NHL that had failed conventional therapy or for which there were no therapeutic alternatives, or for which conventional therapy was not desired by the owner. To be eligible for the study, each dog must have been definitely diagnosed via cytology or histopathology and had met all of the inclusion criteria and none of the exclusion criteria. Additional eligibility criteria included: >1 year old at study entry; adequate organ function; at least 2 weeks since prior chemotherapy or radiation with complete recovery from the acute toxicities of these treatments (3 weeks for a surgical procedure); at least 1 week since prior treatment with any other investigational drug; and no evidence of brain metastases or any serious systemic disorder incompatible with the study at the discretion of the investigator.

### Study Design

This study was a Phase I dose escalating, open label assessment of the safety and biologic activity of KPT-335 in client owned dogs with spontaneous malignancies. Assessment of clinical toxicities and tumor response was performed at each visit. Dogs were evaluated for hematologic and biochemical toxicities every 7 days with routine bloodwork. The initial dose of 1 mg/kg orally twice per week (Monday/Thursday or Tuesday/Friday) was based on previous data from normal laboratory dogs (data not shown) and dose escalation was set at 0.25 mg/kg increments in cohorts of 3 until dose limiting toxicity (DLT) was identified. The DLT was considered to be any grade 3 or 4 hematologic or non-hematologic toxicity based on the established VCOG-CTCAE criteria [36]. Additionally, any chronic non-grade 3 or 4 toxicities considered to significantly impair quality of life (i.e., lethargy, inappetence) were qualified as DLTs. Disease progression or signs and symptoms definitely related to disease were not considered adverse events (AEs). The maximum tolerated dose (MTD) was considered to be one dose below that at which DLT occurred.

### Toxicity Assessment

Each patient underwent a baseline complete history, physical examination, and pre-dose laboratory assessment that included a complete blood count (CBC), serum biochemistry profile, coagulation parameters (PT/PTT) and urinalysis. Patients were assessed for adverse events on days 7, 14, 21, and 28, and every 2 weeks thereafter at which time all laboratory assessments were repeated. Stipulations regarding minimal hematological requirements to continue dosing were included in the protocol: hematocrit >25%, neutrophils >1500/L, platelets >100,000/L. In addition, liver transaminases were required to be <4X upper limit of normal with a normal total bilirubin and serum creatinine to continue KPT-335 therapy.

### Concomitant Medications

To treat drug-related gastrointestinal toxicities, supportive care was administered as needed to dogs enrolled in this study. This typically consisted of famotidine, omeprazole, metronidazole, loperamide, metoclopramide, ondansetron, and/or maropitant. Antihistamines were administered to dogs with mast cell tumors, as these tumors are known to release histamine. Other supportive care administered to dogs consisted of prednisone and non-steroidal anti-inflammatory medications to treat tumor-associated inflammation, inappetence, and for pain control.

### Tumor Response Assessment

Tumor assessments were completed prior to study entry, and days 7, 14, 21, and 28. For dogs that continued beyond 4 weeks, response assessments were performed every 2 weeks thereafter, or at the time of suspected tumor progression. Responses were assessed by the investigator according to pre-defined protocol criteria. The response in dogs with assessable disease was performed by clinical examination, ultrasonography, or thoracic radiography. Many lesions were not amenable for quantitative radiographic imaging, but were followed either by serial clinical examination (superficial lesions; palpable lymph nodes) or by ultrasonography (abdominal lymph nodes). Thoracic lesions were assessed by thoracic radiography.

The response in dogs with measurable disease was judged by the investigator on the basis of Response Evaluation Criteria for Peripheral Nodal Lymphoma in dogs (v1.0) [37]. A complete response (CR) was defined as disappearance of all disease on two measurements separated by a minimum period of 3 weeks. A partial response (PR) was defined as greater than 30% reduction in the sum of the longest diameter of the target lesions documented by two assessments separated by at least 3 weeks. An increase of >20% in the size of all measurable tumor areas as measured by the sum of longest diameters of the target lesions taken as reference the smallest sum since initiation of therapy, or the appearance of any new lesion(s) would qualify as progressive disease (PD). Stable disease (SD) was defined by the absence of criteria for either a response or progression; to be considered SD, dogs must have demonstrated no evidence of PD at the 28 day assessment. Dogs who had no evidence of tumor progression and who had not experienced any unacceptable toxicity were eligible for extended treatment cycles. Dose escalation from twice per week administration to three times per week administration in an individual dog was permitted if the dog was tolerating therapy.

### Quality of Life Assessment

A tool to assess quality of life in dogs with cancer during treatment has been previously published [38]. This was used to assess owner perceived changes in dogs undergoing KPT-335 treatment during both the dose escalation and dose expansion studies. An overall quality of life (QOL) score was created based on answers to the questions on the quality of life questionnaire. The scores from each question were summed resulting in an overall quality of life score which could range from 23 to 115. Trends in Quality of Life (QOL) during the study were examined using linear mixed models containing fixed effects of time and an autoregressive correlation structure for the random errors. Wald tests were used to test for a significant change in QOL with degrees of freedom calculated using the Kenward-Roger method [39]. Measurements obtained after 90 days were not included in the analysis for the dose escalation study and measurements after 70 days were not included in the analysis of the dose expansion data due to few data points after those time points.

## Results

### Activity of SINE Compounds against Canine Tumor Cells *in vitro*

**Figure 1:**
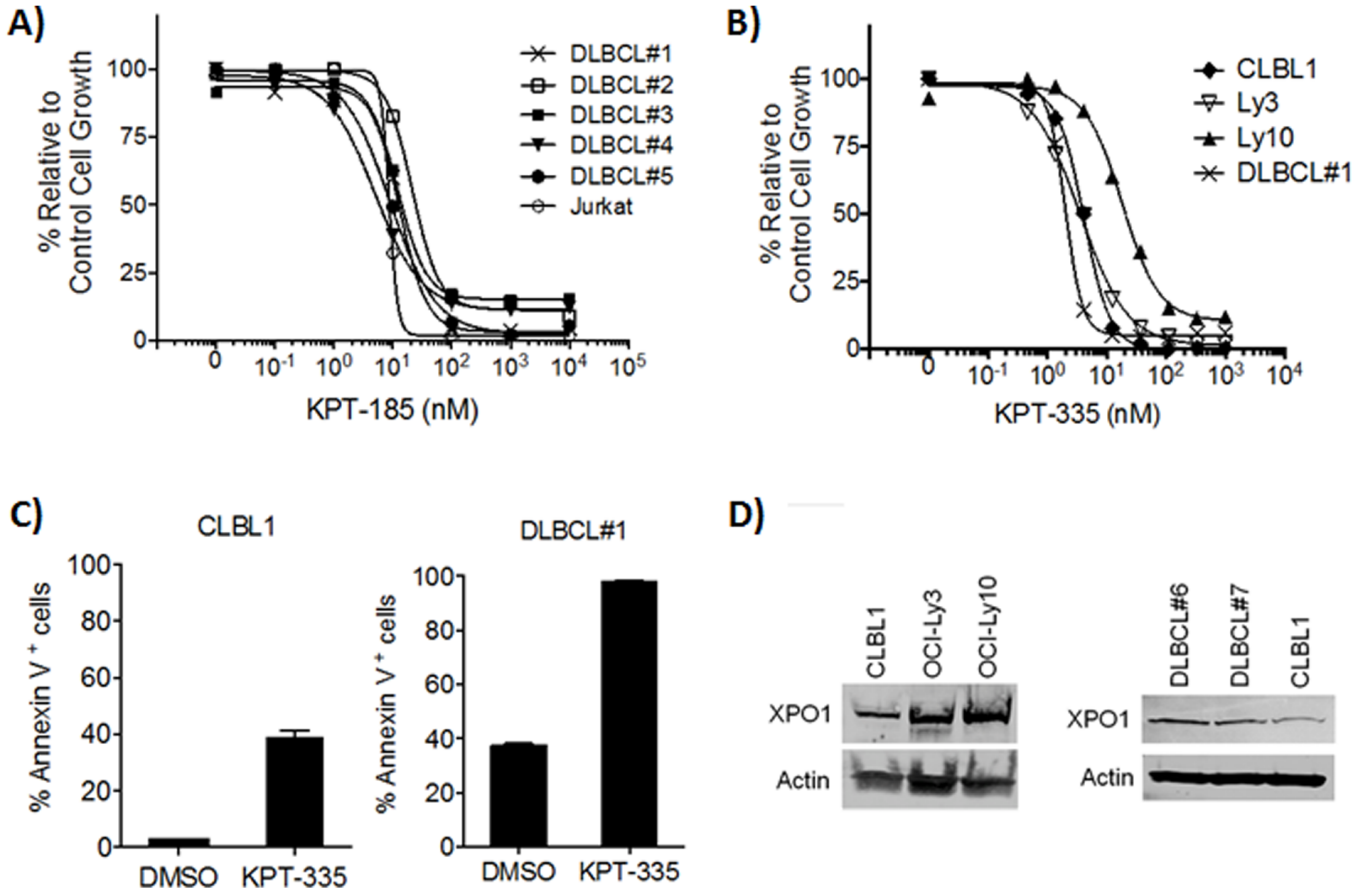
Biologic activity of SINE compounds against canine NHL cells. (A) Jurkat cells and primary canine DLBCL cells (sample #1–5, #5 was independently tested twice) were cultured in 96-well plates for 72 hours with log serial dilutions of KPT-185 and the cell viability was analyzed using the MTS assay. Each experiment was performed in duplicate wells and experiments were repeated three times (B) Human and canine DLBCL cells were cultured in a 96-well plate for 72 hours with 3-fold serial dilutions (0–1000 nM) of KPT-335 and analyzed using the MTS assay. Each experiment was performed in duplicate wells and experiments were repeated three times. (C) CLBL1 cells and primary canine DLBCL cells (sample #1) were treated with 100 nM KPT-335 for 24 hours and analyzed for apoptosis using flow cytometry. Experiments were performed three times independently and the average results are shown. (D) Expression of XPO1 in human and canine DLBCL cell lines. Protein lysates prepared from OCI-Ly3, OCI-Ly10, and CLBL1 were separated by SDS-PAGE and subjected to immunoblotting for XPO1; β-actin served as the control.

We first evaluated the effect of KPT-185, which is one of the most potent SINE compounds but with limited oral bioavailability and therefore only suitable for the *in vitro* studies, on Jurkat (human T cell leukemia) cells and 6 primary canine DLBCL samples (5 distinct tumors with one pair of the same tumor). KPT-185 effectively inhibited the viability of Jurkat and canine DLBCL cells (Figure 1A and Table 1). The IC_50_ of KPT-185 against Jurkat (n = 3) and primary canine DLBCL samples (n = 6) were 8.7±0.7 nM and 13.3±6.2 nM, respectively. KPT185-trans isomer (which has ∼100 fold less XPO1 inhibition activity as the cis-isomer), showed much less toxicity when it was tested using Jurkat cells and one of primary canine DLBCL samples (IC_50_ were >1000 nM in both tumor cells). Next, we evaluated the effect of KPT-335, a clinical candidate compound with good oral exposure used in this clinical trial, on canine and human DLBCL cells. KPT-335 inhibited the viability of OCI-Ly3, OCI-Ly10, and CLBL1 at the IC_50_ of 2.1±1.3 nM, 41.8±21.0 nM, and 8.5±4.1 nM, respectively (Figure 1B and Table 1). We also demonstrated KPT-335 induced apoptosis in CLBL1 cells and primary canine DLBCL cells using flow cytometry. Treatment of cells with KPT-335 for 24 hours increased apoptotic cells (Annexin V^+^ cells) compared to mock treated cells (Figure 1C). Finally, we confirmed the expression of XPO1, which was detected by the estimated size of XPO1 (∼123 kDa), in dog cells using the CLBL1 cell line, human DLBCL cell lines, and canine primary DLBCL cells (Figure 1D). Taken together, both human and canine DLBCL express XPO1 and SINE compounds show potent activity against these tumor cells, suggesting a potential therapeutic benefit for human and canine patients with DLBCL.

**Table 1:** IC_50_ (± S.D.) of SINE for human and canine lymphoma cells.

	KPT-335	KPT-185	KPT-185 trans
Jurkat	0.3	8.7±0.7	>1000
OCI-Ly3	2.1±1.3	24.1	NP
OCI-Ly10	41.8±21.0	246.2	NP
CLBL1	8.5±4.1	NP	NP
Canine DLBCLs	–	13.3±6.2	–
DLBCL#1	2.0	13.1	NP
DLBCL#2	NP	9.0	NP
DLBCL#3	NP	12.2	NP
DLBCL#4	NP	4.9	NP
DLBCL#5	NP	21.6	>1000

IC_50_, 50% inhibitory concentration; DLBCL, diffuse large B-cell lymphoma;

NP, not performed.

**Figure 2:**
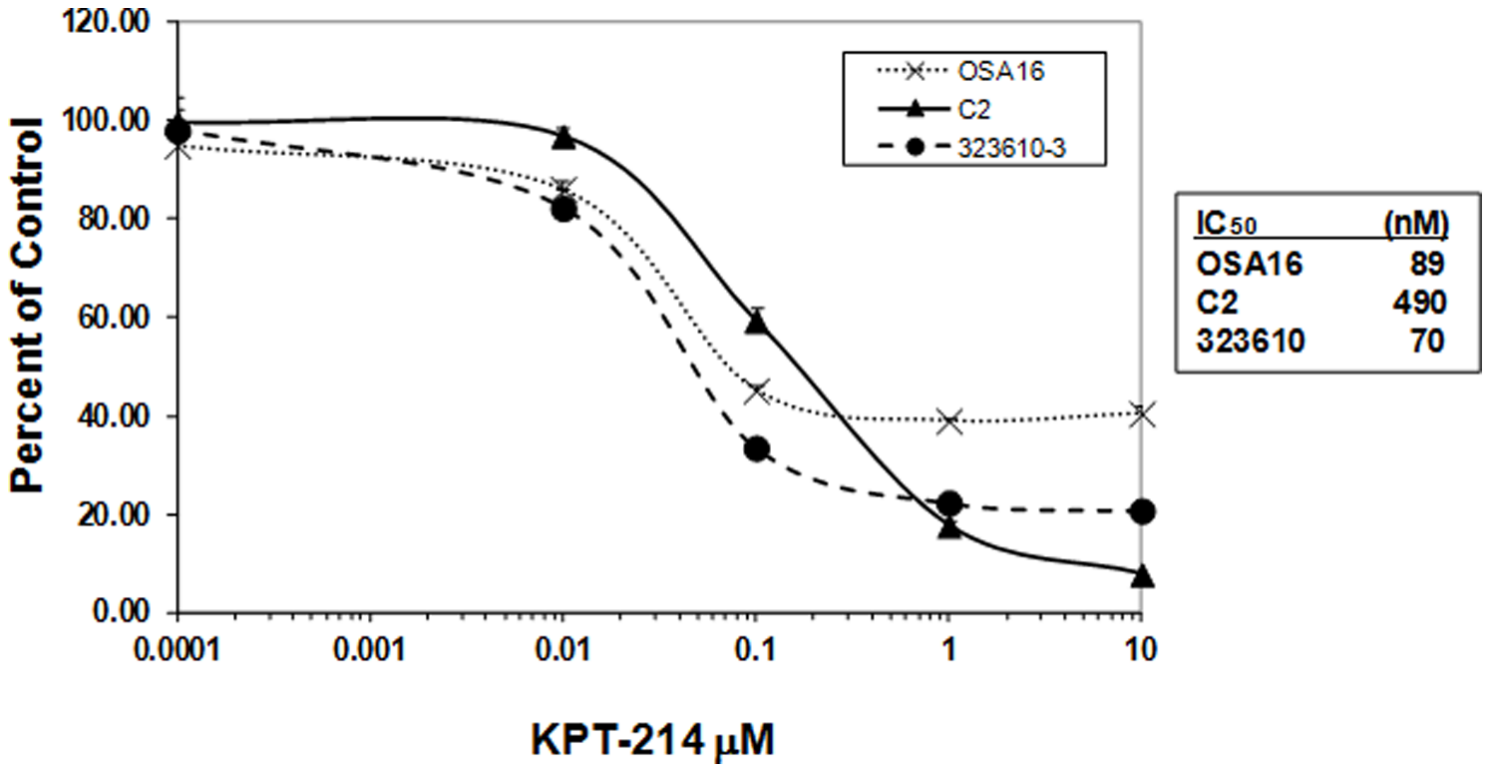
Biologic activity of SINE compounds against canine tumor cell lines. Canine tumor cell lines C2 (mast cell), OSA16 (osteosarcoma) and 323610-3 were cultured in 96 well plates in triplicate with serial dilutions of KPT-214 for 92 hours after which the plates were collected, media removed, and the plates were frozen at −80°C. Analysis for effects on cell proliferation was then performed using the CyQUANT assay according to the manufacturer’s specifications. Experiments were repeated three times; the IC_50_ for each cell line is shown.

To provide a preliminary assessment of the potential activity of SINE XPO1 antagonists against additional canine cancers, the C2 mast cell tumor line, the OSA16 osteosarcoma cell line, and the 323610-3 melanoma cell line were utilized. For these studies an earlier analog, KPT-214, was available for the in vitro experiments (Figure 2). The IC_50_ of KPT-214 against the C2, OSA16 and 323610 were 490 nM, 89 nM, and 70 nM, respectively. In summary, the SINE exhibit biologic activity *in vitro* against a wide variety of canine tumor cell lines, with lymphoid lines being the most sensitive in the very low nanomolar range. These data supported the subsequent *in vivo* evaluation of SINE in dogs with spontaneous cancers, with a particular emphasis on dogs NHL.

### Pharmacokinetics of KPT-335 in Healthy Dogs

To assess the pharmacokinetics of KPT-335, six healthy beagle dogs were administered a single dose of drug at 1.5 mg/kg after being fed a meal. Blood samples were obtained over a 48 hour time period and analyzed for plasma KPT-335 concentrations. The results are shown in Table 2. The mean T_max_ was approximately 4 hours with a C_max_ of approximately 250 ng/ml, and an average AUC of 1800 ng/ml.

**Table 2:** Pharmacokinetics of KPT-335 in healthy dogs.

Parameter	KPT-335 at 1.5 mg/kg
**Dose (mg/kg)**	
Mean	1.46
SD	0.0542
SEM	0.0221
**C_max_ (ng/mL)**	
Mean	253
SD	88.3
SEM	36.1
**T_max_ (hr)**	
Mean	3.83
SD	2.71
SEM	1.11
**t_1/2_ (hr)**	
Mean	3.88
SD	1.47
SEM	0.602
AUC_0-∞_ (h*ng/mL)	
Mean	1810
SD	216
SEM	88.2
AUC_0-last_ (h*ng/mL)	
Mean	1760
SD	223
SEM	90.9

### Dose Escalation Study

#### Patient demographics

Patient demographics and tumor types are listed in Table 3. A total of 17 dogs were enrolled into the dose escalation portion of the study. The median age was 7.5 years and the median weight was 35 kg. The majority of dogs enrolled had NHL (n = 14), and most (n = 12) had also received prior therapy including surgery, chemotherapy and/or prednisone. Prednisone was administered to 10 dogs during the course of KPT-335 treatment at 0.5 mg/kg either once per day or every other day; in 8/10 cases, the dogs entered the study after having experienced disease progression in the face of prednisone therapy and consequently, the drug was not discontinued. The remaining two dogs were placed on prednisone after the first 28 days of treatment to address inappetence/anorexia associated with treatment. These dogs received prednisone at 0.5 mg/kg every other day for the duration of treatment with KPT-335.

**Table 3:** Subject demographics.

Characteristics	Dose Escalation	Dose Expansion
**Number of dogs**	17	6
**Age (yrs)**		
Median	7.5	6.75
Mean	7.7	6.4
Range	4–11	4.5–8
**Weight (kgs)**		
Median	32	23.95
Mean	31.5	20.83
Range	6.2–66.7	4.5–31.4
**Gender**		
Male intact	1	0
Male neutered	8	3
Female intact	1	0
Female neutered	7	3
**Tumor Type**		
Lymphoma	14	6
Mast cell tumor	2	N/A
Osteosarcoma	1	N/A
**Prior Treatment**		
Yes	5	4
No	12	2
**Prednisone**		
Yes	10	6
No	7	0

#### Clinical toxicities and maximum tolerated dose

For all dogs enrolled in the dose escalation portion of the study, the primary clinical toxicities consisted of mainly grade 1 and 2 gastrointestinal events including anorexia, vomiting and diarrhea, as well as lethargy (summarized in Tables 4 and 5). The MTD was established as 1.75 mg/kg given twice per week (Monday/Thursday). All three dogs in the 2 mg/kg cohort experienced DLT including grade 3 anorexia (n = 2), grade 3 vomiting (n = 1), grade 3 diarrhea (n = 1), grade 3 ALT elevation (n = 2), grade 3 AST elevation (n = 1), and grade 3 ALP elevation (n = 1). Therefore, the adverse event profile of KPT-335 was mainly limited to the gastrointestinal tract and clinically relevant hepatotoxicity was observed mainly at doses above the MTD.

**Table 4:** Constitutional and gastrointestinal toxicities.

Dose (mg/kg)	No. of dogs	Lethargy				Anorexia				Weight Loss				Vomiting				Diarrhea			
		1	2	3	4	1	2	3	4	1	2	3	4	1	2	3	4	1	2	3	4
1.0	3	1				2	1			3				1				1			
1.25	3	2				3	1			2					1						
1.5	5	3	1			3	2			5	2			2				1		1	
1.75	3	2	1			1	1			2	2			1				1		1	
2.0	3		1				1	2		3	2			1	2	1				1	
1.5 MWF	6	3				7	1	1		4	2			3	1			3	1		

**Table 5:** Hepatic and hematologic toxicities.

Dose (mg/kg)	No. of dogs	ALP				ALT				Bilirubin				Anemia				Thrombo-cytopenia			
		1	2	3	4	1	2	3	4	1	2	3	4	1	2	3	4	1	2	3	4
1.0	3	4	2			1								2	1			2	1		
1.25	3	1				2								3	1			1			
1.5	5	4		1		3		1			1			3	2			3			
1.75	3	1	1	1		1	1							1	1						
2.0	3	2	1	1		2	1	2		1					1	2					
1.5 MWF	6	2		1	1	4	2	1		1				1				1	1		

In 4 cases, dogs that were on drug for over 4 weeks had a change in regimen from twice per week to three times per week (Monday/Wednesday/Friday) as drug was well tolerated and the dogs were experiencing stable disease. This occurred in one dog receiving 1 mg/kg, two dogs receiving 1.25 mg/kg, and one dog receiving 1.5 mg/kg. They remained on this increased dosing frequency regimen for the duration of therapy (median 68.5 days) without any increase in clinical toxicities. In 4 cases dose modifications were made for dogs that experienced adverse events. Two of these occurred in dogs that received drug at 2 mg/kg and experienced grade 3 adverse events; in both instances, the dose was reduced to 1.5 mg/kg twice per week. In the other two instances, one dog was reduced from 1.75 mg/kg to 1.5 mg/kg then 1.25 mg/kg due to continuing issues with anorexia, and the other dog was reduced from 1.5 mg/kg to 1.25 mg/kg, also due to anorexia. This last patient remained on therapy for 246 days.

#### Response to therapy

The median TTP for all dogs was 35 days (range 14–246 days). A total of 7 dogs experienced PD in the first 4 weeks of therapy. Two dogs had a PR for 71 and 246 days, and 8 dogs experienced SD for a median of 58.5 days (range 28–84 days). Of these 10 dogs, 6 were receiving prednisone prior to starting KPT-335 that continued during treatment and 4 did not receive prednisone during their treatment. All but one of the dogs with clinical benefit (CB) associated with KPT-335 administration (PR or SD >4 weeks) had NHL with a median TTP in responding dogs of 66 days (range 35–256 days).

### Dose Expansion Study

#### Patient demographics

Based on the results of the dose escalation study and responses to therapy observed in dogs with NHL, an additional 6 dogs with NHL received KPT-335 at 1.5 mg/kg given on a Monday/Wednesday/Friday schedule. Patient demographics are again provided in Table 3. The median age was 6.75 years and the median weight was 23.95 kg. Four of the dogs had previously received treatment for their NHL (primarily multi-agent chemotherapy), and all were receiving prednisone as part of their therapy, with demonstrated progression of disease prior to study entry. These dogs were maintained on prednisone at 0.5 mg/kg either daily or every other day during KPT-335 administration. Two other dogs had not received any prior therapy and prednisone at 0.5 mg/kg given every other day was administered to alleviate anorexia/inappetence after 28 days on study for the duration of KPT-335 treatment.

#### Clinical toxicities

As with the dose escalation portion of the study, the most common toxicities were gastrointestinal in nature and primarily grade 1 and 2 in severity, including anorexia, weight loss, vomiting and diarrhea (summarized in Tables 4 and 5). The grade 3 adverse events consisted of anorexia (n = 1), increased ALT (n = 1, not clinically relevant), and increased ALP (n = 2, most likely related to ongoing prednisone administration); one dog had a grade 4 ALP elevation, again most likely related to prednisone therapy.

#### Response to therapy

The median TTP for all dogs was 55 days (range 13–354 days). Two dogs had a PR for 35 and 354 days, and 4 dogs experience SD for longer than 28 days, for a CB rate of 67% (4/6 dogs) with a median TTP of 83 days (range 35–354 days).

### Quality of Life Assessment

**Figure 3:**
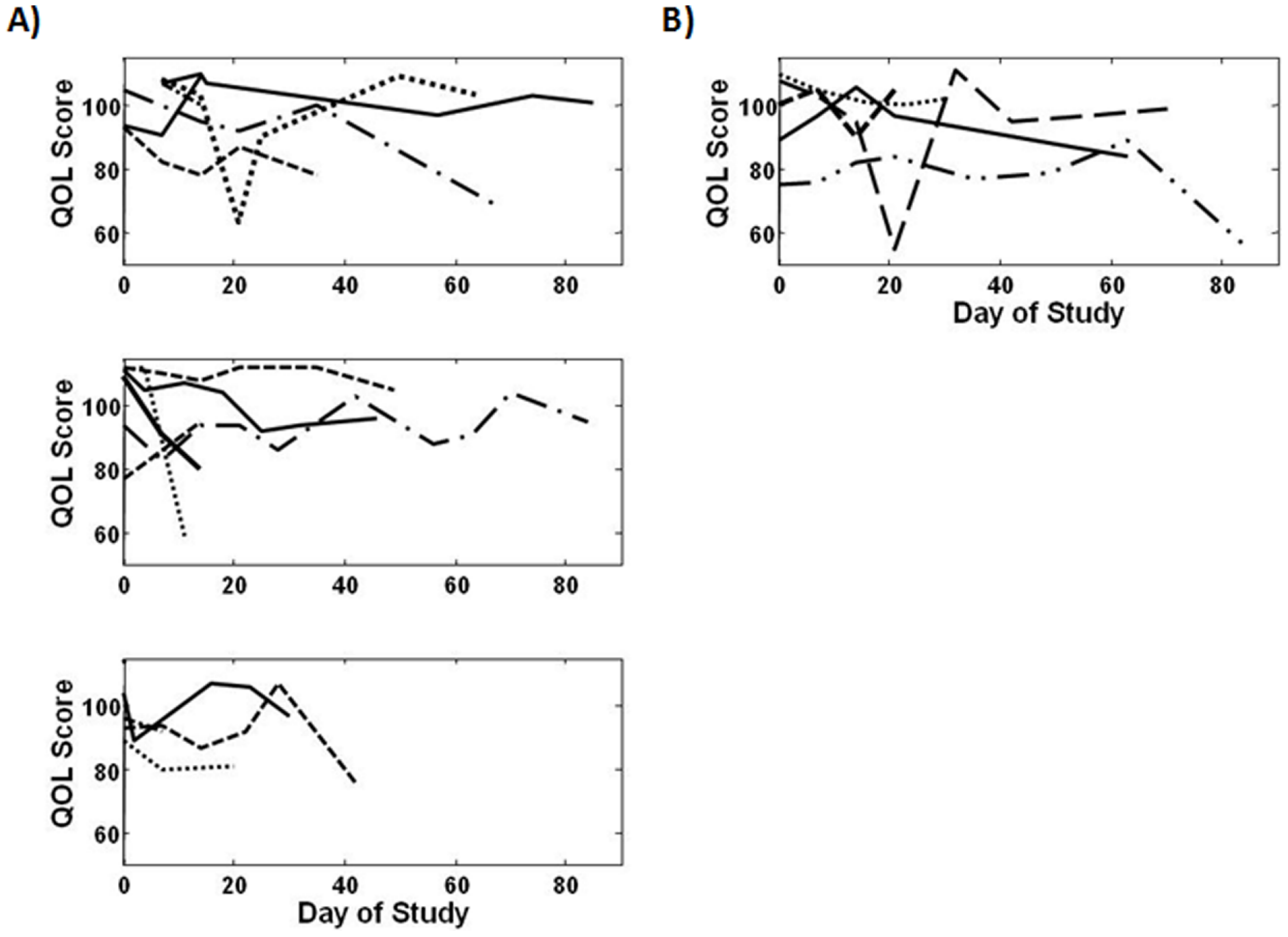
Trends in quality of life in dogs treated with KPT-335. An overall score was created based on answers to questions on the quality of life questionnaire. The scores from each question were summed resulting in an overall quality of life score which could range from 23 to 115. These are represented graphically in the figure above where scores for each patient are graphed over time (each line represents a patient). Trends in quality of life during the study were examined using linear mixed models. The overall quality of life did not change significantly in dogs treated in either the (A) dose escalation study (p = 0.64) or (B) dose expansion study (p = 0.47).

Dog owners were asked to complete a health related Quality of Life (QOL) form prior to study entry and at each recheck visit [38]. As shown in Figure 3, the overall quality of life did not change significantly in dogs treated in either the dose escalation study (p = 0.64) or dose expansion study (p = 0.47). These data support the notion that clinical toxicities associated with KPT-335 did not decrease quality of life in treated dogs.

## Discussion

XPO1 is responsible for the nuclear-cytoplasmic export of over 200 proteins [4], [40] and several RNAs, many of which mediate pathways that control proliferation and survival. Several studies have now demonstrated that adequate function of XPO1 is necessary for cancer cells to survive [2], [3]. Interestingly, XPO1 inhibition appears to have selective effects primarily on cancer cells, while sparing normal cells in most cases [19]. The mechanisms responsible for this differential effect are not entirely clear. The majority of tumor suppressor proteins (TSPs) such including p53, p73, p21, p27, Rb and FOXO are exported from the nucleus exclusively by XPO1 [7], [41]. Inhibition of XPO1 results in forced nuclear retention, upregulation, and activation of TSPs [1], [2], [3]. Restoration of TSP function can induce apoptosis in cells with a damaged genome, including tumor cells. Since XPO1 inhibition leads to the restoration of so many TSPs at once, it is theorized that anti-tumor activity can be independent of oncogenic drivers responsible for maintenance of the neoplastic phenotype [42], [43]. It has also been suggested that when TSPs localize to the nucleus, a genome survey is initiated that tumor cells fail. In contrast, XPO1 inhibition in normal cells typically results in a transient cell cycle arrest in the absence of cell death and subsequent recovery once inhibition has been relieved. This phenomenon was demonstrated *in vitro* where inhibition of XPO1 did not induce cytotoxicity in normal B, T and NK cells [15], [19].

While several small molecule inhibitors of XPO1 have been previously developed, only a few have been tested in mouse models. Despite its marked toxicities in animals, the natural product Leptomycin B is the sole XPO1 inhibitor that was tested in human clinical trials prior to the development of the SINE compounds. Consistent with side effects identified in mice, a Phase I study in people induced severe anorexia and malaise while providing no evidence of biologic activity prohibiting its further clinical development [10]. The semi-synthetic Leptomycin B derivative KOS-2464 with improved pharmacological parameters compared with Leptomycin B, exhibited significant activity against several tumor cell lines *in vitro*, while sparing normal cells. KOS-2464 given intravenously was active in all mouse xenograft models tested while inducing significantly less weight loss than Leptomycin B [11]. An oral small molecule reversible inhibitor of XPO1, CBS9106 induced cell cycle arrest and apoptosis in 60 different human tumor cell lines and suppressed tumor growth in mouse xenografts without any significant morbidity or mortality [44]. Both KOS-2464 and CBS9106 have not yet been evaluated in human clinical trials.

SINE compounds are a series of orally bioavailable, slowly reversible inhibitors of XPO1 that demonstrate high specificity for target, based on covalent binding to the reactive cysteine 528 residue in the cargo binding domain [12], [15], [17], [19]. Several of the early analogs have been evaluated *in vitro* and in mouse models of cancer and all have shown excellent biologic activity with minimal toxicity. KPT-185 and KPT-276 were shown to be active against AML cell lines and primary blasts from patients, and significantly prolonged the survival of leukemic mice in a FLT3-ITD+ AML xenograft model [14], [17]. Similar results were obtained with KPT-251 in a CLL mouse model [15]. Activity of KPT-185 and KPT-276 was demonstrated against MCL, follicular lymphoma and DLBCL both *in vitro* and in mouse models of disease [18], [45]. Inhibition of XPO1 using KPT-185, -251, -276, and -330 impaired melanoma survival in both BRAF mutant and wild-type melanoma cell lines and in mouse xenografts [46]. Furthermore, SINE compounds exhibited synergistic activity with BRAF inhibitors PLX4720 and PLX4032 against BRAF mutant melanoma [46]. Lastly, several of the SINE compounds including KPT-330 have shown activity against pancreatic cell tumor lines i*n vitro* and slowed tumor growth of subcutaneous and orthotopic xenograft tumors [12]. Importantly, all of the compounds tested exhibited a good safety profile in the mouse models.

The purpose of the current study was first to evaluate the effect of SINE compounds in canine tumor cell lines, and then second, to assess KPT-335 in a spontaneous model of cancer that would provide important information regarding dose, regimen, and clinical toxicity profile thereby laying the groundwork for subsequent human clinical trials. Our data demonstrate that similar to the case of human cancer cell lines, KPT-185, KPT-214 and the clinical candidate KPT-335 all demonstrate inhibition of cell proliferation and induction of apoptosis in multiple canine tumor cell lines, with particularly strong activity against canine NHL cells. This was anticipated as the sequence (Cys528 equivalent) and binding pockets of XPO1 from Schizosaccharomyces pombe up to humans are highly conserved [1], [2], [3]. In canine NHL lines, biologic effects were noted in the low nanomolar range with IC_50_ concentrations typically below 20 nM. This is concordant with the effects of other SINE compounds against human AML, MCL and CLL cell lines. Additionally, we found that KPT-214 was active against canine mast cell tumor, osteosarcoma, and melanoma cell lines, also at nanomolar drug concentrations; studies are ongoing to evaluate the biologic activity of KPT-335 against canine melanoma cell lines and inhibition of cell proliferation and viability has been observed at IC_50_ concentrations ranging from 95–290 nM (data not shown). Based on these data and information generated from evaluation of KPT-335 administration to normal dogs, a Phase I study was initiated in dogs with spontaneous cancers including NHL, mast cell cancer, metastatic osteosarcoma and malignant melanoma.

For the clinical trial in dogs with cancer, KPT-335 was used as this compound exhibits excellent oral bioavailability and good pharmacokinetic properties in normal laboratory dogs. In this setting, doses below 3 mg/kg given either twice per week or three times per week (Monday/Thursday or Monday/Wednesday/Friday) were found to be tolerable, with 2 mg/kg considered to be the MTD for chronic administration (data not shown). Given this information, KPT-335 dosing for the Phase I study was initiated at 1 mg/kg Monday/Thursday. DLT were observed at 2 mg/kg and as such the maximum tolerated dose was established as 1.75 mg/kg. However, it is important to note that biologic activity was observed at all dose levels including 1 mg/kg, with the dose of 1.25-1.5 mg/kg Monday/Thursday initially established as the range tolerable over chronic dosing periods.

While dogs with NHL, mast cell tumor, osteosarcoma, and melanoma were eligible to receive KPT-335 during the Phase I dose escalation, the majority of dogs actually entered had NHL (14/17). Within this group, 9/14 (64%) exhibited evidence of clinical benefit (2 PR, 7 SD for greater than 28 days) following KPT-335 administration and the median duration of treatment for dogs with NHL was 10 weeks (range 5–35 weeks). Importantly, biologic activity of KPT-335 was observed in dogs with NHL that had not been previously treated as well as in dogs that relapsed or were refractory to standard chemotherapy treatment (typically a CHOP based regimen) and therefore were considered drug resistant. The dose limiting toxicities observed consisted primarily of gastrointestinal events (anorexia, weight loss, vomiting, and diarrhea) and at 2 mg/kg, drug related hepatotoxicity (elevated ALT, bilirubin). Although the hepatotoxicity did not result in obvious clinical effects and rapidly resolved following drug discontinuation, KPT-335 did induce loss of appetite that was moderately refractory to standard anti-emetic therapy consisting of metoclopramide, ondansetron, and/or maropitant. Inappetence associated with minimal emesis has been observed with other animal species, and anorexia is consistently observed across all species, suggesting that it may be a mechanism-based toxicity. Most of the dogs enrolled in this study had been receiving prednisone prior to study entry and while this did appear to enhance appetite, it did not completely resolve the toxicity in all dogs.

The majority of dogs were dosed twice per week on a Monday/Thursday basis, however a few dogs (n = 4) were switched to a Monday/Wednesday/Friday regimen during ongoing therapy. Based on these data and the observed biologic activity of KPT-335 in dogs with NHL, an expansion study was undertaken using the three times per week regimen. We elected to use the dose of 1.5 mg/kg rather than the MTD of 1.75 mg/kg as we had observed biologic activity at doses as low as 1 mg/kg and anticipated having fewer challenges with anorexia and weight loss. Clinical benefit of KPT-335 treatment occurred in 4/6 dogs treated using this regimen, with responding dogs on drug for over 8 weeks (range 5–52). The Monday/Wednesday/Friday regimen was associated with mainly grade 1 and 2 gastrointestinal toxicities (anorexia, weight loss, vomiting, and diarrhea) that did not result in drug discontinuation, although 3 dogs did undergo a dose reduction to 1.25 mg/kg while on treatment.

As previously discussed, the primary barriers to clinical development of previous XPO1 small molecule inhibitors have consisted of marked anorexia and malaise observed in both mice and humans. These toxicities have been viewed as a drug class effect, expected to occur with all inhibitors that block XPO1 function. While anorexia was identified as one of the major dose limiting events associated with KPT-335 administration, it was deemed manageable when drug was administered between 1–1.5 mg/kg on a 2 or 3 times per week basis. Indeed, several dogs (n = 10) remained on drug for 8 weeks or longer (range 9–52) indicating that KPT-335 is not only associated with biologic activity in canine NHL, but can be well tolerated over chronic administration. This is further supported by the fact that quality of life scores, a key indicator of how dog owners perceive their dogs’ response KPT-335 therapy, did not diminish over time.

One of the limitations of this clinical trial was the potential confounding use of prednisone in most of the dogs with NHL treated with KPT-335 and its potential impact on response to therapy. In both the dose escalation and dose intensification portions of the study, approximately half of dogs with NHL were receiving prednisone prior to study entry (11/20). For those that experienced either PR or SD (for greater than 28 days), 7/13 had experienced disease progression while on prednisone prior to study entry, and the drug was not discontinued. The decision to maintain prednisone use in these dogs was multifactorial. Several of the dogs had been receiving drug for a long period of time and would need to have the prednisone tapered over 2–3 weeks prior to study entry. Additionally, given the potential issues with anorexia, it was felt that continuing prednisone in the dogs already being given drug might help to ameliorate this toxicity. With respect to the remaining 6 dogs with NHL that experience CB, 4 of these were placed on low dose prednisone after 28+ days of treatment to address anorexia. The remaining 2 dogs with CB never received prednisone as part of their therapy. Therefore, 9/13 dogs with CB from KPT-335 treatment either had progression in the face of prednisone therapy or were not given prednisone during the course of the clinical trial supporting the notion that the observed biologic activity of KPT-335 was at least in part due to XPO1 inhibition.

In summary, this phase I clinical trial represents the first evaluation of a novel oral XPO1 inhibitor in a spontaneous large animal model of cancer. Our data demonstrate that the administration of KPT-335 results in an acceptable and tolerable spectrum of clinical toxicities over prolonged dosing periods without impairment of quality of life. Furthermore, KPT-335 treatment contributed to either objective response to therapy or prolonged disease stabilization in dogs with NHL, supporting the notion that, as demonstrated in mouse models of disease, XPO1 inhibition has biologic activity in lymphoid malignancies. Given the marked similarities between canine NHL and human NHL, these data provide critical new information that has direct applicability for the evaluation of SINE compounds in humans with hematologic and other cancers.

## References

[1] Nguyen KT, Holloway MP, Altura RA (2012) The CRM1 nuclear export protein in normal development and disease. Int J Biochem Mol Biol 3: 137–151. 22773955 PMC3388738

[2] Turner JG, Dawson J, Sullivan DM (2012) Nuclear export of proteins and drug resistance in cancer. Biochem Pharmacol 83: 1021–1032. 22209898 10.1016/j.bcp.2011.12.016 PMC4521586

[3] Turner JG, Sullivan DM (2008) CRM1-mediated nuclear export of proteins and drug resistance in cancer. Curr Med Chem 15: 2648–2655. 18991627 10.2174/092986708786242859

[4] Xu D, Grishin NV, Chook YM (2012) NESdb: a database of NES-containing CRM1 cargoes. Mol Biol Cell 23: 3673–3676. 22833564 10.1091/mbc.E12-01-0045 PMC3442414

[5] Guttler T, Gorlich D (2011) Ran-dependent nuclear export mediators: a structural perspective. EMBO J 30: 3457–3474. 21878989 10.1038/emboj.2011.287 PMC3181476

[6] Daelemans D, Costes SV, Lockett S, Pavlakis GN (2005) Kinetic and molecular analysis of nuclear export factor CRM1 association with its cargo in vivo. Mol Cell Biol 25: 728–739. 15632073 10.1128/MCB.25.2.728-739.2005 PMC543413

[7] Fornerod M, Ohno M, Yoshida M, Mattaj IW (1997) CRM1 is an export receptor for leucine-rich nuclear export signals. Cell 90: 1051–1060. 9323133 10.1016/s0092-8674(00)80371-2

[8] Tai YT, Landesman Y, Acharya C, Calle Y, Zhong MY, et al. (2013) CRM1 inhibition induces tumor cell cytotoxicity and impairs osteoclastogenesis in multiple myeloma: molecular mechanisms and therapeutic implications. Leukemia Epub ahead of print. 10.1038/leu.2013.115 PMC3883926 23588715

[9] Aloisi A, Di Gregorio S, Stagno F, Guglielmo P, Mannino F, et al (2006) BCR-ABL nuclear entrapment kills human CML cells: ex vivo study on 35 patients with the combination of imatinib mesylate and leptomycin B. Blood. 107: 1591–1598. 10.1182/blood-2005-05-2123 16249386

[10] Newlands ES, Rustin GJ, Brampton MH (1996) Phase I trial of elactocin. Br J Cancer 74: 648–649. 8761384 10.1038/bjc.1996.415 PMC2074658

[11] Mutka SC, Yang WQ, Dong SD, Ward SL, Craig DA, et al (2009) Identification of nuclear export inhibitors with potent anticancer activity in vivo. Cancer Res 69: 510–517. 19147564 10.1158/0008-5472.CAN-08-0858 PMC2635062

[12] Azmi AS, Aboukameel A, Bao B, Sarkar FH, Philip PA, et al (2012) Selective Inhibitors of Nuclear Export Block Pancreatic Cancer Cell Proliferation and Reduce Tumor Growth in Mice. Gastroenterology 144: 447–456. 23089203 10.1053/j.gastro.2012.10.036 PMC3594519

[13] Draetta GG, Shacham S, Kauffman M, Sandanayaka V, Schechter S, et al (2011) Cytotoxicity of novel, small molecule, CRM1-selective inhibitors of nuclear export (SINE) in colorectal cancer (CRC) cells. J Clin Oncol 29: e14091.

[14] Etchin J, Sanda T, Mansour MR, Kentsis A, Montero J, et al (2013) KPT-330 inhibitor of CRM1 (XPO1)-mediated nuclear export has selective anti-leukaemic activity in preclinical models of T-cell acute lymphoblastic leukaemia and acute myeloid leukaemia. Br J Haematol 161: 117–127. 23373539 10.1111/bjh.12231 PMC3980736

[15] Lapalombella R, Sun Q, Williams K, Tangeman L, Jha S, et al (2012) Selective inhibitors of nuclear export show that CRM1/XPO1 is a target in chronic lymphocytic leukemia. Blood 120: 4621–4634. 23034282 10.1182/blood-2012-05-429506 PMC3512237

[16] McCauley D, Landesman Y, Senapedis W, Kashyap T, Saint-Martin J-R, et al. (2012) Preclinical evaluation of selective inhibitors of nuclear export (SINE) in basal-like breast cancer (BLBC). J Clin Oncol 30 Suppl: Abstract 1055.

[17] Ranganathan P, Yu X, Na C, Santhanam R, Shacham S, et al (2012) Preclinical activity of a novel CRM1 inhibitor in acute myeloid leukemia. Blood 120: 1765–1773. 22677130 10.1182/blood-2012-04-423160 PMC3433086

[18] Zhang K, Wang M, Tamayo AT, Shacham S, Kauffman M, et al. (2013) Novel selective inhibitors of nuclear export CRM1 antagonists for therapy in mantle cell lymphoma. Exp Hematol 41: 67–78 e64. 10.1016/j.exphem.2012.09.002 22986101

[19] Etchin J, Sun Q, Kentsis A, Farmer A, Zhang ZC, et al (2013) Antileukemic activity of nuclear export inhibitors that spare normal hematopoietic cells. Leukemia 27: 66–74. 22847027 10.1038/leu.2012.219 PMC3542631

[20] Inoue H, Kauffman M, Shacham S, Landesman Y, Yang J, et al (2013) CRM1 Blockade by Selective Inhibitors of Nuclear Export Attenuates Kidney Cancer Growth. J Urol 189: 2317–2326. 23079374 10.1016/j.juro.2012.10.018 PMC4593314

[21] London CA, Hannah AL, Zadovoskaya R, Chien MB, Kollias-Baker C, et al (2003) Phase I dose-escalating study of SU11654, a small molecule receptor tyrosine kinase inhibitor, in dogs with spontaneous malignancies. Clin Cancer Res 9: 2755–2768. 12855656

[22] Pryer NK, Lee LB, Zadovaskaya R, Yu X, Sukbuntherng J, et al (2003) Proof of target for SU11654: inhibition of KIT phosphorylation in canine mast cell tumors. Clin Cancer Res 9: 5729–5734. 14654558

[23] Vail DM, Thamm DH, Reiser H, Ray AS, Wolfgang GH, et al (2009) Assessment of GS-9219 in a pet dog model of non-Hodgkin’s lymphoma. Clin Cancer Res 15: 3503–3510. 19417014 10.1158/1078-0432.CCR-08-3113

[24] Honigberg LA, Smith AM, Sirisawad M, Verner E, Loury D, et al (2010) The Bruton tyrosine kinase inhibitor PCI-32765 blocks B-cell activation and is efficacious in models of autoimmune disease and B-cell malignancy. Proc Natl Acad Sci U S A 107: 13075–13080. 20615965 10.1073/pnas.1004594107 PMC2919935

[25] London CA, Bear MD, McCleese J, Foley KP, Paalangara R, et al (2011) Phase I Evaluation of STA-1474, a Prodrug of the Novel HSP90 Inhibitor Ganetespib, in Dogs with Spontaneous Cancer. PLoS One 6: e27018. 22073242 10.1371/journal.pone.0027018 PMC3207826

[26] Ito D, Frantz AM, Williams C, Thomas R, Burnett RC, et al (2012) CD40 ligand is necessary and sufficient to support primary diffuse large B-cell lymphoma cells in culture: a tool for in vitro preclinical studies with primary B-cell malignancies. Leuk Lymphoma 53: 1390–1398. 22229753 10.3109/10428194.2011.654337 PMC3727651

[27] Jubala CM, Wojcieszyn JW, Valli VE, Getzy DM, Fosmire SP, et al (2005) CD20 expression in normal canine B cells and in canine non-Hodgkin lymphoma. Vet Pathol 42: 468–476. 16006606 10.1354/vp.42-4-468

[28] Rutgen BC, Hammer SE, Gerner W, Christian M, de Arespacochaga AG, et al (2010) Establishment and characterization of a novel canine B-cell line derived from a spontaneously occurring diffuse large cell lymphoma. Leuk Res 34: 932–938. 20153049 10.1016/j.leukres.2010.01.021

[29] Rutgen BC, Willenbrock S, Reimann-Berg N, Walter I, Fuchs-Baumgartinger A, et al (2012) Authentication of primordial characteristics of the CLBL-1 cell line prove the integrity of a canine B-cell lymphoma in a murine in vivo model. PLoS One 7: e40078. 22761949 10.1371/journal.pone.0040078 PMC3386195

[30] Yee C, Biondi A, Wang XH, Iscove NN, de Sousa J, et al (1989) A possible autocrine role for interleukin-6 in two lymphoma cell lines. Blood 74: 798–804. 2787680

[31] Yee CS, Messner HA, Minden MD (1991) Regulation of interleukin-6 expression in the lymphoma cell line OCI-LY3. J Cell Physiol 148: 426–429. 1918171 10.1002/jcp.1041480314

[32] Gururajan M, Chui R, Karuppannan AK, Ke J, Jennings CD, et al (2005) c-Jun N-terminal kinase (JNK) is required for survival and proliferation of B-lymphoma cells. Blood 106: 1382–1391. 15890690 10.1182/blood-2004-10-3819 PMC1895189

[33] DeVinney R, Gold WM (1990) Establishment of two dog mastocytoma cell lines in continuous culture. Am J Respir Cell Mol Biol 3: 413–420. 2121170 10.1165/ajrcmb/3.5.413

[34] McMahon MB, Bear MD, Kulp SK, Pennell ML, London CA (2010) Biological activity of gemcitabine against canine osteosarcoma cell lines in vitro. Am J Vet Res 71: 799–808. 20594083 10.2460/ajvr.71.7.799

[35] Shelly S, Chien MB, Yip B, Kent MS, Theon AP, et al (2005) Exon 15 BRAF mutations are uncommon in canine oral malignant melanomas. Mamm Genome 16: 211–217. 15834638 10.1007/s00335-004-2441-x

[36] (2011) Veterinary cooperative oncology group - common terminology criteria for adverse events (VCOG-CTCAE) following chemotherapy or biological antineoplastic therapy in dogs and cats v1.1. Vet Comp Oncol Epub ahead of print. 10.1111/vco.283 28530307

[37] Vail DM, Michels GM, Khanna C, Selting KA, London CA (2010) Response evaluation criteria for peripheral nodal lymphoma in dogs (v1.0)–a Veterinary Cooperative Oncology Group (VCOG) consensus document. Vet Comp Oncol 8: 28–37. 20230579 10.1111/j.1476-5829.2009.00200.x

[38] Lynch S, Savary-Bataille K, Leeuw B, Argyle DJ (2011) Development of a questionnaire assessing health-related quality-of-life in dogs and cats with cancer. Vet Comp Oncol 9: 172–182. 21848620 10.1111/j.1476-5829.2010.00244.x

[39] Kenward MG, Roger JH (1997) Small sample inference for fixed effects from restricted maximum likelihood. Biometrics 53: 983–997. 9333350

[40] Xu D, Farmer A, Collett G, Grishin NV, Chook YM (2012) Sequence and structural analyses of nuclear export signals in the NESdb database. Mol Biol Cell 23: 3677–3693. 22833565 10.1091/mbc.E12-01-0046 PMC3442415

[41] Fukuda M, Asano S, Nakamura T, Adachi M, Yoshida M, et al (1997) CRM1 is responsible for intracellular transport mediated by the nuclear export signal. Nature 390: 308–311. 9384386 10.1038/36894

[42] Martins CP, Brown-Swigart L, Evan GI (2006) Modeling the therapeutic efficacy of p53 restoration in tumors. Cell 127: 1323–1334. 17182091 10.1016/j.cell.2006.12.007

[43] Ventura A, Kirsch DG, McLaughlin ME, Tuveson DA, Grimm J, et al (2007) Restoration of p53 function leads to tumour regression in vivo. Nature 445: 661–665. 17251932 10.1038/nature05541

[44] Sakakibara K, Saito N, Sato T, Suzuki A, Hasegawa Y, et al (2011) CBS9106 is a novel reversible oral CRM1 inhibitor with CRM1 degrading activity. Blood 118: 3922–3931. 21841164 10.1182/blood-2011-01-333138

[45] Azmi AS, Al-Katib A, Aboukameel A, McCauley D, Kauffman M, et al (2013) Selective inhibitors of nuclear export for the treatment of non-Hodgkin’s lymphomas. Haematologica 98: 1098–1106. 23403316 10.3324/haematol.2012.074781 PMC3696614

[46] Salas Fragomeni RA, Chung HW, Landesman Y, Senapedis W, Saint-Martin JR, et al (2013) CRM1 and BRAF inhibition synergize and induce tumor regression in BRAF mutant melanoma. Mol Cancer Ther 12: 1171–1179. 23615632 10.1158/1535-7163.MCT-12-1171

